# Associations between BRAF^V600E^ and prognostic factors and poor outcomes in papillary thyroid carcinoma: a meta-analysis

**DOI:** 10.1186/s12957-016-0979-1

**Published:** 2016-09-06

**Authors:** Chunping Liu, Tianwen Chen, Zeming Liu

**Affiliations:** 1 Department of Breast and Thyroid Surgery, Union Hospital, Tongji Medical College, Huazhong University of Science and Technology, Number 1277, Jiefang Road, Wuhan, Hubei Province China; 2 Department of Breast and Thyroid Surgery, Renmin Hospital of Wuhan University, Number 238, Jiefanglu, Wuhan, Hubei Province People’s Republic of China; 3 Department of Breast and Thyroid Surgery, Affiliated Nanshan Hospital, Guangdong Medical College, Number 89, Taoyuan Road, Shenzhen, China

**Keywords:** Papillary thyroid carcinoma, BRAF mutation, Meta-analysis

## Abstract

**Background:**

The objective of this study is to perform a meta-analysis to evaluate the associations between the BRAF^V600E^ mutation status and aggressive clinicopathological features and poor prognostic factors in papillary thyroid cancer.

**Methods:**

A literature search was performed within the PubMed, MEDLINE, Web of Science databases, and EMBASE databases using the Medical Subject Headings and keywords from January 2003 to July 2015. Individual study-specific odds ratios and confidence intervals were calculated, as were the Mantel-Haenszel pooled odds ratios for the combined studies.

**Results:**

Sixty-three studies of 20,764 patients were included in the final analysis. Compared with wild-type BRAF, the BRAF^V600E^ mutation was associated with aggressive clinicopathological factors, including extrathyroidal extension, higher TNM stage, lymph node metastasis, and recurrence, and was associated with reduced overall survival; however, there was no significant association between the presence of BRAF mutation and distant metastasis.

**Conclusions:**

BRAF mutations are closely associated with aggressive clinicopathological characteristics and poorer prognosis in papillary thyroid cancer. Accordingly, aggressive treatment should be considered for papillary thyroid cancer patients with BRAF mutation.

## Background

Papillary thyroid carcinoma (PTC) is the most common malignant thyroid neoplasm, accounting for 80 % of all thyroid cancers [1]. The incidence of PTC has increased dramatically in the past decades [2], and Chen et al. demonstrated that the increasing rate of PTC resulted from an actual increase in disease incidence rather than improvements in the diagnosis such as by high-resolution ultrasound [3]. PTCs, especially papillary thyroid microcarcinomas, tend to have a good prognosis; however, even for TNM stage I patients, approximately 15 % of patients experienced recurrence during 10 years of follow-up in one previous study [4]. Therefore, the classification and targeted therapies for PTC still need more investigation.

The BRAF^V600E^ mutation is a common mutation in PTC, with a reported frequency of 25–82.3 %. Many authors have reported that the BRAF^V600E^ mutation is associated with aggressive clinical features and poor prognosis; however, others have suggested that there is no association between the BRAF^V600E^ mutation and clinicopathologic features in PTCs [5–7]. Thus, it remains controversial whether the BRAF^V600E^ mutation closely correlates with poor clinical characteristics in PTC, and it is necessary to confirm the association between BRAF^V600E^ and the clinical prognosis of PTC using meta-analysis.

Accordingly, the present meta-analysis was designed to determine whether the BRAF^V600E^ mutation is associated with high-risk clinicopathological factors and poor prognostic outcomes in PTC patients. Additionally, we performed subgroup analyses to assess the effects of factors that might modify these associations. The results of our meta-analysis are considered very helpful for clinical surgeons to choose the optimal surgical managements, such as whether prophylactic central neck dissection is needed, and for postoperative risk stratification of PTCs.

## Methods

A comprehensive search was conducted in the PubMed, MEDLINE, and Web of Science databases from January 2003 to July 2015. The search terms and keywords used included “papillary thyroid carcinoma,” “BRAF,” “mutation,” “V600E,” “T1799A,” and “PTC.” All studies that included BRAF^V600E^ mutation data from primary PTC tissues were further evaluated. In addition, the reference lists from single case reports and relevant reviews articles were also inspected for further eligible studies by two independent reviewers. Clinical characteristics such as extrathyroidal invasion, lymph node metastasis, TNM stage, cancer recurrence, and rates of cancer-specific death at the last follow-up were included in the present analysis. The search was restricted to English language publications only. Duplicate articles and studies with no clinicopathologic data were excluded. To avoid false-negative results, studies that determined the BRAF^V600E^ mutation status by preoperative fine needle aspiration biopsies only were also excluded. Further, studies confined to only low-risk groups or papillary thyroid microcarcinomas were excluded, as were unpublished data that were presented at international meetings. In instances where the same study cohort was used in multiple articles, either the most recent or the most appropriately informative single article was included. For example, Fugazzola et al. [6] included only data from a single institute and contained a cohort overlapping with Fugazzola et al. [8]; therefore, we used the latter study for our analysis.

### Data analyses and statistical methods

We used RevMan (version 5) to calculate the summary odds ratios (ORs) with 95 % confidence intervals (CIs), using a random-effects model when *p* value (chi-square test of homogeneity) <0.1 and a fixed-effects model when *p* value (chi-square test of homogeneity) ≥0.1. We assessed the heterogeneity of the studies using the chi-square test of heterogeneity and the *I*^2^ measure of inconsistency. Significant heterogeneity was defined as a chi-square test *p* value of <0.10 or as an *I*^2^ measure >50 % (according to a statement from Cochrane Handbook). The extent to which the combined metarisk or heterogeneity were affected by individual studies was assessed further by sequentially excluding every study from the meta-analysis. We investigated potential sources of the identified heterogeneity among the studies using a stratification process according to the country or ethnics in which the research was conducted. The potential for publication bias was assessed using a funnel plot analysis.

## Results

### Results of the literature search and study characteristics

**Fig. 1:**
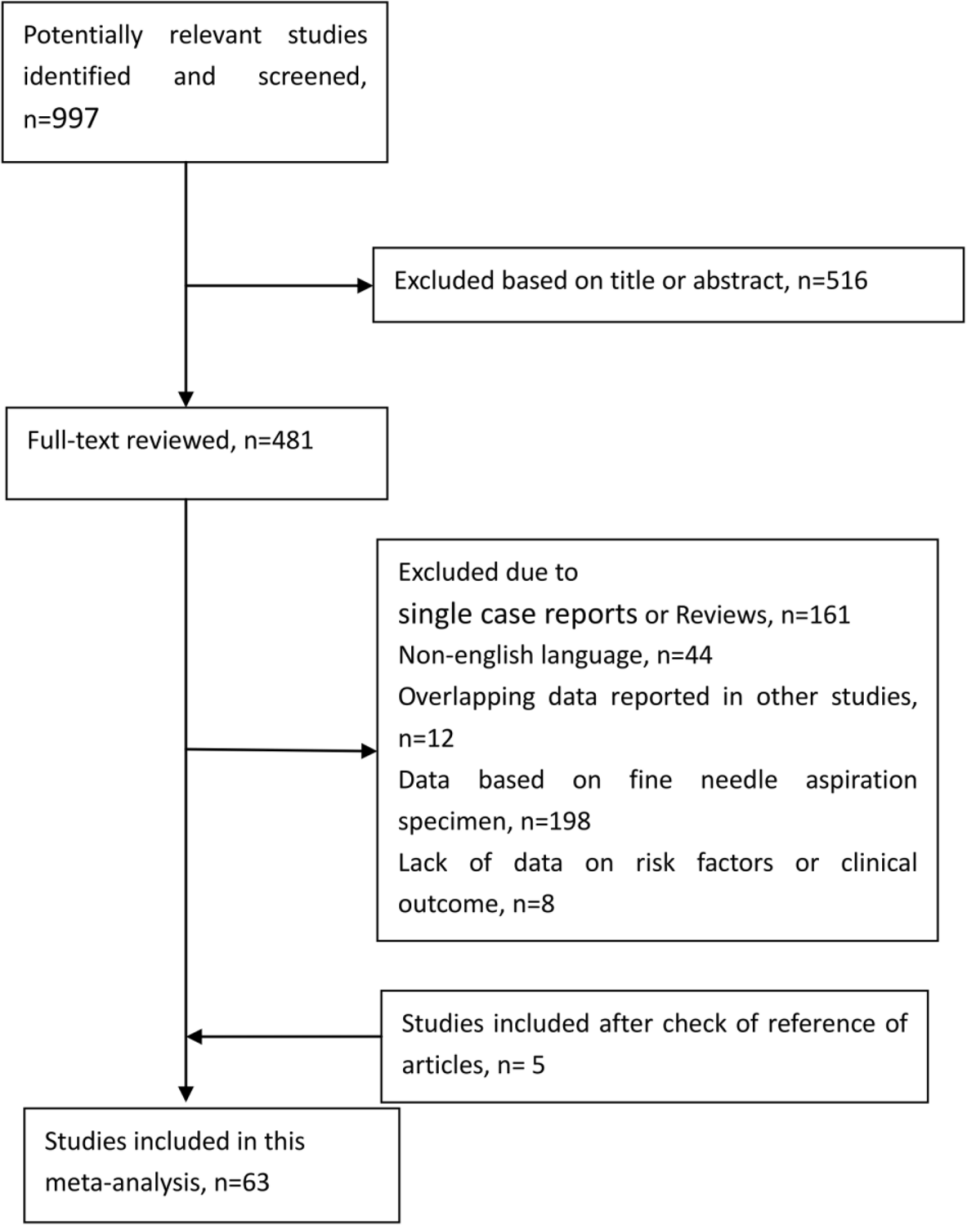
The study selection process

Figure 1 shows the study selection process. A total of 997 abstracts and titles of full-text papers obtained through electronic searches were deemed relevant and examined in detail. Following this detailed review, 63 studies met our inclusion/exclusion criteria; these studies contributed 20,764 patients with PTC to this meta-analysis [6, 8–69]. The main features of the 63 eligible studies that investigated prognostic factors are summarized in Table 1. The funnel plots for each outcome did not suggest the presence of publication bias (data not shown).

**Table 1:** Summary of the 63 included studies

Study	Ethnic A: Asia C: Caucasia	PTC number positive/negative	BRAFV600E mutation rate, %
O'Neill [26]	A	85/22	79.4
Daliri [65]	A	28/41	40.6
Han [51]	A	353/146	70.7
HE [59]	A	119/68	63.6
Namba [28]	A	26/14	65.0
Ahn [27]	A	38/88	30.2
Hong1 [61]	A	120/73	62.2
Hong2 [61]	A	1792/639	73.7
Liu [12]	A	213/126	62.8
Jung [36]	A	162/48	77.1
Kim [41]	A	64/15	81.0
Kim [7, 43]	A	34/69	33.0
Kim [7]	A	149/54	73.4
Kim [66]	A	265/57	82.3
LEE [16]	A	58/42	42.0
Lee [9]	A	24/40	37.5
Lim [54]	A	2219/728	75.3
Liu [40]	A	47/54	46.5
Liu [12]	A	80/52	60.6
Rivera [42]	A	43/18	70.5
Khan [56]	A	15/45	25
Shao [58]	A	133/67	66.5
Wang [57]	A	80/35	69.6
Lee [16]	A	241/390	38.4
Puxeddu [22]	A	281/485	36.4
Yim [50]	A	123/41	75
Jo [44]	A	102/59	63.4
Zeng [62]	A	465/154	75.1
Henke [67]	A and C	340/168	66.9
Czarniecka [64]	C	38/50	43.2
Abrosimov [34]	C	23/17	57.5
Alzahrani [47]	C	96/185	34.2
Guerra [48]	C	90/78	53.6
Frasca [25]	C	60/41	59.4
Ahn [27]	C	217/286	45.4
Durante [20]	C	56/37	60.2
Nikiforova [21]	C	24/36	40.0
Frasca [25]	C	125/198	38.7
Fugazzola [8]	C	99/161	38.1
Basolo [32]	C	472/575	45.1
Basolo [32]	C	229/349	39.6
Oler [49]	C	48/25	65.8
Howell [14]	C	86/133	39.3
Nakayama [29]	C	173/124	58.2
Fernandez [30]	C	153/143	51.7
Fugazzola [6]	C	18/29	38.3
Lee [69]	C	44/19	70
Li [55]	C	297/91	76.5
Yip [39]	C	99/100	49.7
Jung [36]	C	19/24	44.2
Durante [20]	C	38/66	36.5
Howell [14]	C	15/40	27.3
Pelizzo [35]	C	98/43	69.5
McKelvie [68]	C	45/22	67.2
Riesco [37]	C	28/39	41.8
Ito [23]	C	38/64	37.3
Russo [60]	C	57/46	55.3
Ulisse [45]	C	44/47	48.4
Sykorova [63]	C	81/161	33.5
Musholt [46]	C	122/168	42.1
Angell [53]	C	16/17	48.9
Xing [18]	C	107/112	48.9
Xing [19]	C	73/117	40.6
Xing [52]	C	194/313	38.3
Xing [1, 10]	C/A	845/1004	45.7

### Meta-analyses of *BRAF* mutation effects on clinicopathological and prognostic features

#### BRAF mutation and extrathyroidal extension

**Fig. 2:**
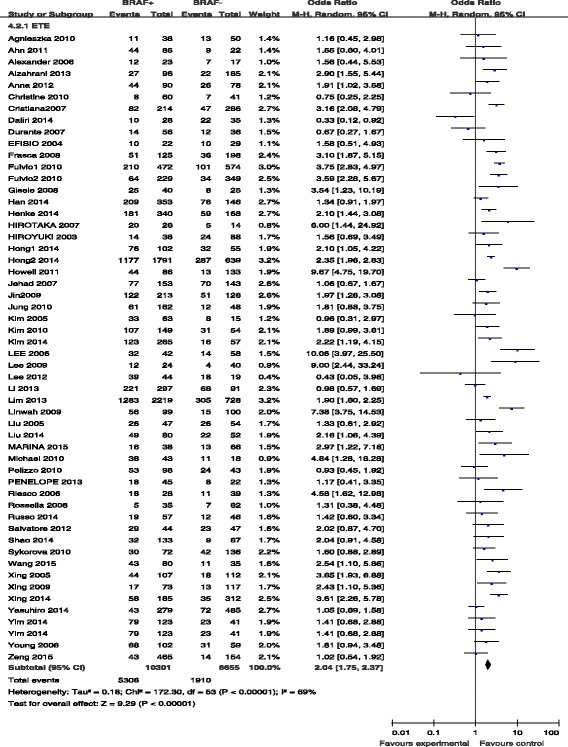
The odds ratios (ORs) with 95 % confidence intervals (CIs) for the association between BRAF mutation and extrathyroidal extension in patients with PTC

Fifty-two studies contained data on extrathyroidal invasion. Extrathyroidal invasion was present in 5306 (51.5 %) of 10,301 patients with BRAF mutations and in 1910 (28.7 %) of 6655 patients without BRAF mutations (Fig. 2). The pooled odds ratio (OR) from these 52 studies was 2.04 (95 % confidence interval [CI], 1.75–2.37). A random-effects model was adopted because the heterogeneity of the data was significant (*p* < 0.00001), and the *I*^2^ estimate of the variance between the studies was 69 %. According to our analysis, the association between the occurrence of extrathyroidal invasion and *BRAF* mutation was significant (*p* < 0.00001).

### BRAF mutation and TNM stage

**Fig. 3:**
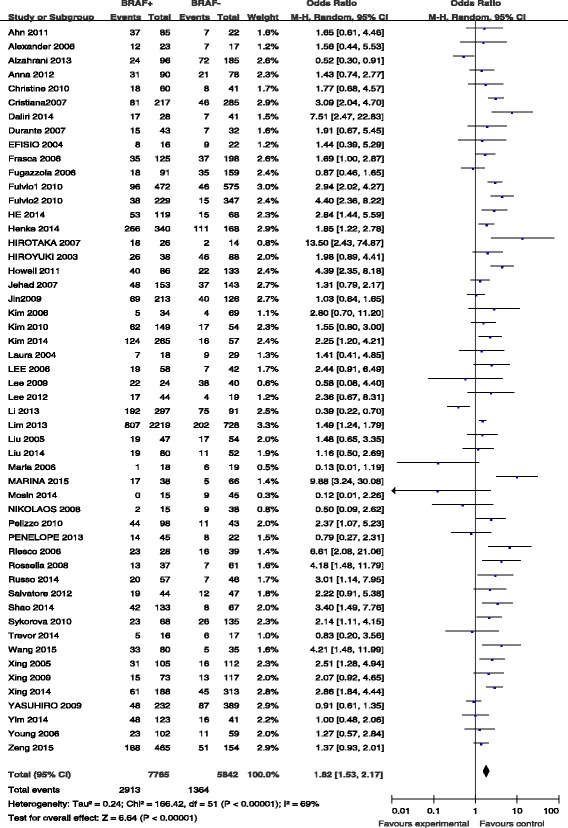
The odds ratios (ORs) with 95 % confidence intervals (CIs) for the association between BRAF mutation and advanced TNM stage (III/IV) in patients with PTC

TNM stage was reported for patients in 51 studies (Fig. 3). The advanced TNM stage (III/IV) was present in 2913 (37.5 %) of 7765 patients with BRAF mutations and in 1364 (23.3 %) of 5842 patients without BRAF mutations (Fig. 3). The pooled odds ratio (OR) from these 51 studies was 1.82 (95 % CI, 1.53–2.17). A random-effects model was adopted because the heterogeneity of the data was significant (*p* < 0.00001), and the *I*^2^ estimate of the variance between the studies was 70 %. According to our analysis, the association between the occurrence of advanced TNM stage (III/IV) and *BRAF* mutations was significant (*p* < 0.00001).

### BRAF mutation and lymph node metastasis

**Fig. 4:**
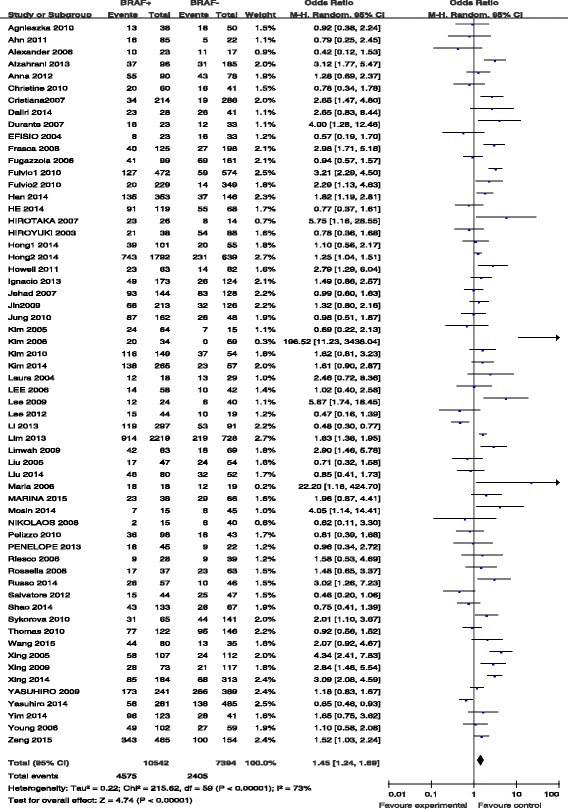
The odds ratios (ORs) with 95 % confidence intervals (CIs) for the association between BRAF mutation and lymph node metastasis in patients with PTC

Lymph node metastasis was reported for patients in 51 studies (Fig. 4). Lymph node metastasis was present in 4575 (43.4 %) of 10,542 patients with BRAF mutations and in 2405 (32.5 %) of 7394 patients without BRAF mutations (Fig. 4). The pooled odds ratio (OR) from these 51 studies was 1.45 (95 % CI, 1.24–1.69). A random-effects model was adopted because the heterogeneity of the data was significant (*p* < 0.00001), and the *I*^2^ estimate of the variance between the studies was 73 %. According to our analysis, the association between the occurrence of lymph node metastasis and *BRAF* mutations was significant (*p* < 0.00001).

### BRAF mutation and distant metastasis

**Fig. 5:**
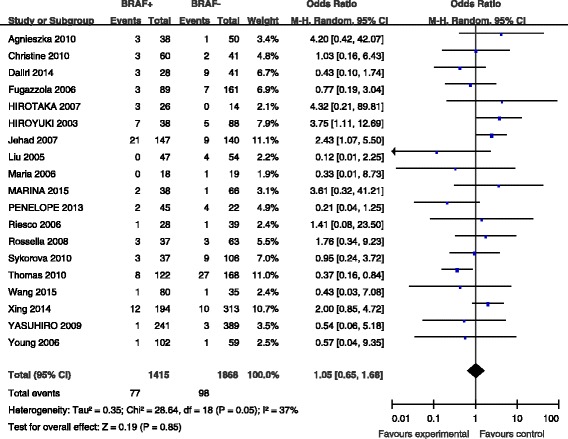
The odds ratios (ORs) with 95 % confidence intervals (CIs) for the association between BRAF mutation and distant metastasis in patients with PTC

Distant metastasis was reported for patients in 19 studies (Fig. 5). Distant metastasis was present in 77 (5.4 %) of 1415 patients with BRAF mutations and in 98 (5.2 %) of 1868 patients without BRAF mutations (Fig. 5). The pooled odds ratio (OR) from these 19 studies was 1.05 (95 % CI, 0.65–1.68). A random-effects model was adopted because the heterogeneity of the data was significant (*p* = 0.05), and the *I*^2^ estimate of the variance between the studies was 37 %. According to our analysis, the association between the occurrence of distant metastasis and BRAF mutations was not significant (*p* = 0.85).

### BRAF mutation and recurrence

**Fig. 6:**
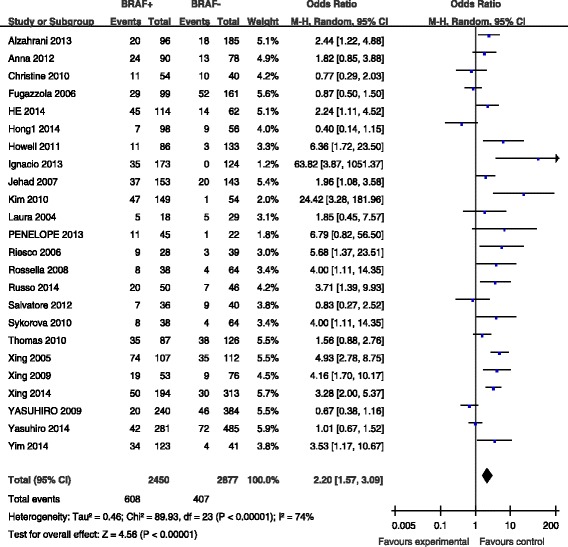
The odds ratios (ORs) with 95 % confidence intervals (CIs) for the association between BRAF mutation and recurrence with PTC

Recurrence was reported in 24 studies (Fig. 6). Recurrence was present in 608 (24.8 %) of 2450 patients with BRAF mutations and in 407 (14.1 %) of 2877 patients without BRAF mutations (Fig. 6). The pooled OR from these 24 studies was 2.20 (95 % CI, 1.57–3.09). A random-effects model was adopted because the heterogeneity of the data was significant (*p* < 0.00001), and the *I*^2^ estimate of the variance between the studies was 74 %. According to our analysis, the association between the occurrence of recurrence and BRAF mutations was significant (*p* < 0.00001).

### BRAF mutation and OS

**Fig. 7:**
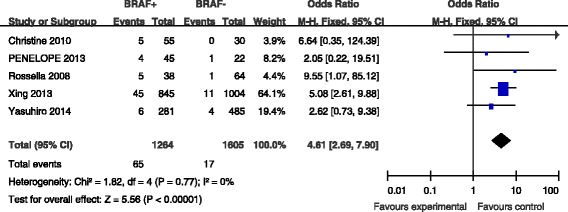
The odds ratios (ORs) with 95 % confidence intervals (CIs) for the association between BRAF mutation and OS with PTC

Overall survival (OS) was reported in five studies (Fig. 7). OS was present in 65 (5.1 %) of 1264 patients with BRAF mutations and in 17 (1.1 %) of 1605 patients without BRAF mutations (Fig. 7). The pooled OR from these five studies was 4.61 (95 % CI, 2.69–7.90). A fixed-effects model was adopted because the heterogeneity of the data was not significant (*p* = 0.77), and the *I*^2^ estimate of the variance between the studies was 0 %. According to our analysis, the association between the occurrence of death and BRAF mutations was significant (*p* = <0.00001).

### Subgroup analyses of the BRAF mutation effects on aggressive clinicopathological features and poor prognostic factors

Subgroup analysis was conducted according to the ethnicities of the study subjects in order to investigate the potential sources of heterogeneity and to assess whether the effects of the BRAF mutation status on aggressive clinicopathological features and poor prognosis of PTC patients were associated with geographic regions (Table 2). The heterogeneity was decreased in the subgroup analyses of extrathyroidal invasion, TNM stage, lymph node metastasis, and recurrence. Thus, the results of the subgroup analyses indicated that the BRAF mutation status may differ according to ethnicity.

**Table 2:** Subgroup analysis of effects of *BRAF* mutation on aggressive clinicopathological features and poor prognosis of PTC according to ethnics

Subgroup	OR	OR (95 % CI)	*I* ^2^ (%)	Model used
Extrathyroidal invasion				
Asian (23 studies)	1.84	1.53–2.22	58	Random-effects
Caucasian (26 studies)	1.19	1.69–2.84	74	Random-effects
TNM stage				
Asian (20 studies)	1.69	1.34–2.14	56	Random-effects
Caucasian (30 studies)	1.72	1.28–2.32	78	Random-effects
Lymph node metastasis				
Asian (27 studies)	1.25	1.06–1.49	58	Random-effects
Caucasian (30 studies)	1.54	1.19–1.98	77	Random-effects
Recurrence				
Asian (6 studies)	1.30	1.01–1.68	80	Random-effects
Caucasian (18 studies)	2.48	2.06–2.98	73	Random-effects

*OR* odds ratio, *RR* risk ratio, *CI* confidence interval

## Discussion

BRAF^V600E^ was first described as a mutation related to the MEK-ERK pathway in human cancer by Davies et al. in 2002. Since then, the BRAF^V600E^ mutation has been reported as a biological marker for the aggressiveness and prognosis of numerous cancers, including malignant melanoma and thyroid cancer among others. Among the three Raf isoforms, BRAF is the strongest downstream activator of the MEK pathway. As a downstream target of MEK, the phosphorylation of ERK can activate substrates located in both the cytoplasm and nucleus. Abnormal activation of this pathway, for example by BRAF mutation, results in the disruption of biological homeostasis and may result in tumor transformation [70, 71].

Since its initial discovery, the BRAF^V600E^ mutation has been debatable as a diagnostic as well as prognostic indicator of PTC, as many of the studies on the topic have reported contradictory outcomes of the association between the BRAF^V600E^ mutation and clinical characteristics and prognosis [37, 40]. Therefore, the present meta-analysis to illustrate the role of BRAF mutation in PTC was considered highly necessary.

Various clinicopathological risk factors have been reported to be related to recurrence and cancer death in PTC [72]; according to most authors’ results, we chose extrathyroidal invasion, lymph node metastasis, advanced TNM stage, and distant metastasis as reliable predictors for poor prognosis in the present analysis, as these factors not only represent aggressive cancer behavior but also poor prognosis [72, 73].

Extrathyroidal extension is associated with an increased risk of invasion into cervical structures such as the trachea, which requires more aggressive treatment. Accordingly, extrathyroidal extension is an important factor related to PTC prognosis, contributing to an increased risk of local recurrence/persistence of the disease, independent of the tumor size [74]. In our meta-analysis, the risk of extrathyroidal extension was increased by 2.04-fold in cases with positive BRAF mutation status compared to in wild-type cases. In fact, out of all included studies, only six studies showed that patients with BRAF mutation presented less frequent extrathyroidal extension than those without BRAF mutation.

The American Joint Committee on Cancer (AJCC) staging system comprises the sum of several tumor characteristics, such as the tumor size, lymph node status, and distant metastasis, which are generally considered aggressive features of cancer. AJCC stage III/IV cancers are associated with a poorer prognosis in terms of both recurrence and overall survival than stage I/II tumors [75]. According to our results, there was a significant association between high AJCC stage and BRAF mutation, with positive BRAF mutation status being associated with a 1.82-fold increased risk of stage III/IV cancers compared to wild-type cases. Out of all included studies, nine studies suggested that the risk of a high AJCC stage was lower in patients with BRAF mutation than in those without BRAF mutation; however, the other 42 studies demonstrated the opposite results, especially for patients aged >45 years, in whom positive BRAF mutation status was associated with more frequent cases of stage III/IV cancers compared to stage I/II [20, 76]. Howell et al. also reported that older age and positive mutation status were more frequently associated with TNM stage III/IV and recurrence [14].

Further, according to the present meta-analysis, the prevalence of lymph node metastasis was increased in patients with BRAF mutation, with an odds ratio of 1.45; increased risk was observed in 39 of 60 studies. Lymph node metastasis is an important risk factor for recurrence and/or persistent disease, as well as overall survival [75]. Based on these results, we suggest that the presence of BRAF mutation is a prognostic factor in PTCs. However, it is worth mentioning that the different surgical approaches and treatments used in these 60 studies probably influenced the outcome in terms of the real incidence of nodal metastasis; therefore, evaluation of the BRAF mutation status as a prognostic indicator for lymph node metastasis in PTCs should be more cautious [74].

Recurrence and persistent disease demand additional therapy and can affect the PTC patients’ quality of life. For example, recurrence increases the risk of reoperations and the exposure to a high cumulative radioiodine dose. Recently, Xing et al., in their large multicenter study, demonstrated that the BRAF^V600E^ mutation was an independent prognostic factor for PTC recurrence in various clinicopathologic categories [77]. However, these outcomes were questioned by Bal & Ballal and Yarchoan et al. [78, 79], as the median follow-up was only 36 months and the recurrence rate was very high, which might have resulted from persistent disease rather than true recurrence. However, our meta-analysis indicated that the association between the occurrence of recurrence and BRAF mutations was significant, and these results were obtained from 24 studies including a total of 2450 patients; hence, our findings are considered more reliable.

Our present meta-analysis encompassing a total of 20,764 patients suggested that the BRAF^V600E^ mutation is associated with several of the high-risk clinical variables used in prognostic staging systems, including extrathyroidal invasion, high TNM stage, lymph node metastasis, recurrence, and overall survival. Especially, PTCs with positive BRAF^V600E^ mutation exhibited a 4.61-fold increased risk of death compared with cases with the wild-type form of the BRAF gene. On the other hand, one interesting finding in the present meta-analysis was that the BRAF^V600E^ mutation was not related to distant metastasis. One potential reason for this result may be the fact that the diagnosis of distant metastasis differs between different countries and medical centers.

There were several limitations regarding our study design, especially in terms of the studies included in our analysis. Additionally, our study could not determine a causal relationship between *BRAF* mutations and outcomes, and *BRAF* mutations may correlate with some other mutations or confounders, which may turn out to be even more useful prognostic indicators. Importantly, our analysis should be interpreted with caution because of the heterogeneity in the data. Possible explanations for this heterogeneity include differences in the patient demographics and ethnicities, as well as in the thyroidectomy, approaches to lymph node dissection, pathology reporting, radioactive iodine treatment, and the time of follow-up. Therefore, our conclusions should be interpreted with caution.

## Conclusions

In conclusion, our meta-analysis illustrated that the BRAF^V600E^ mutation correlates with high-risk clinicopathological factors and poor clinical outcome. These results provide a valuable reference for physicians when assessing the prognosis of PTCs by BRAF^V600E^ mutation analysis, and our findings may be of great value in evidence-based clinical decision-making for PTC patients.

## Abbreviations

AJCC: American Joint Committee on Cancer
PTC: Papillary thyroid carcinoma
